# TIMP-2 Fusion Protein with Human Serum Albumin Potentiates Anti-Angiogenesis-Mediated Inhibition of Tumor Growth by Suppressing MMP-2 Expression

**DOI:** 10.1371/journal.pone.0035710

**Published:** 2012-04-24

**Authors:** Mi-Sook Lee, Jae-In Jung, Seung-Hae Kwon, Sang-Mok Lee, Kyoji Morita, Song Her

**Affiliations:** 1 Division of Bio-Imaging, Chuncheon Center, Korea Basic Science Institute, Chuncheon, Republic of Korea; 2 BiocurePharm, Daejeon Bio Venture Town, Daejeon, Republic of Korea; 3 Laboratory of Neuropharmacology, Department of Nursing, Shikoku University, School of Health Sciences, Tokushima, Japan; Wayne State University School of Medicine, United States of America

## Abstract

TIMP-2 protein has been intensively studied as a promising anticancer candidate agent, but the *in vivo* mechanism underlying its anticancer effect has not been clearly elucidated by previous works. In this study, we investigated the mechanism underlying the anti-tumor effects of a TIMP-2 fusion protein conjugated with human serum albumin (HSA/TIMP-2). Systemic administration of HSA/TIMP-2 effectively inhibited tumor growth at a minimum effective dose of 60 mg/kg. The suppressive effect of HSA/TIMP-2 was accompanied by a marked reduction of *in vivo* vascularization. The anti-angiogenic activity of HSA/TIMP-2 was directly confirmed by CAM assays. In HSA/TIMP-2-treated tumor tissues, MMP-2 expression was profoundly decreased without a change in MT1-MMP expression of PECAM-1-positive cells. MMP-2 mRNA was also decreased by HSA/TIMP-2 treatment of human umbilical vein endothelial cells. Zymographic analysis showed that HSA/TIMP-2 substantially decreased extracellular pro-MMP-2 activity (94–99% reduction) and moderately decreased active MMP-2 activity (10–24% reduction), suggesting MT1-MMP-independent MMP-2 modulation. Furthermore, HSA/TIMP-2 had no effect on *in vitro* active MMP-2 activity and *in vivo* MMP-2 activity. These studies show that HSA/TIMP-2 potentiates anti-angiogenic activity by modulating MMP-2 expression, but not MMP-2 activity, to subsequently suppress tumor growth, suggesting an important role for MMP-2 expression rather than MMP-2 activity in anti-angiogenesis.

## Introduction

Angiogenesis is a fundamental process comprising the development of new blood vessels from preexisting vessels in physiological and pathological conditions. Development of new blood vessels is one predominant mode of tumor progression, thus the therapeutic manipulation of angiogenesis has emerged as a promising strategy for cancer treatment [1], [2], [3]. Although tumor angiogenesis is a complex multistep process, the degradation of components of the extracellular matrix (ECM) by proteolytic enzymes known as matrix metalloproteinases (MMPs) represents a crucial stage [4]. Artificial inhibitors to control ECM proteolysis in angiogenic tumors have been developed and characterized in many laboratory and clinical trials [4], [5], [6]. However, human clinical trials of synthetic MMP inhibitors for the treatment of cancers have failed to show therapeutic benefit in humans [5]. As an alternative, natural inhibitors of MMPs (tissue inhibitors of matrix metalloproteinases; TIMPs) have increasingly attracted attention as promising anticancer candidate agents.

TIMPs have multiple functions in cell proliferation, migration, apoptosis, and differentiation in addition to their MMP-inhibitory activities [6], [7], [8]. TIMP-2, one of four TIMPs, is a key regulator of, and potential therapeutic agent against, angiogenesis and tumor progression [9]. Gene therapy studies using adenoviral and retroviral delivery systems in tumors have demonstrated a role for TIMP-2 in suppressing angiogenesis and subsequent tumor progression in animal models [10], [11]. However, virus-mediated TIMP-2 gene therapy in humans may limit its therapeutic application due to safety concerns, including cytotoxicity and immune responses, along with it being a possible causative agent of cancer [12], [13].

TIMP-2, a non-glycosylated protein, can be expressed in *Escherichia coli*, but this process often results in the formation of insoluble protein aggregates as inclusion bodies. Following the recovery process including denaturation and renaturation, one of the critical points for achieving a biologically active protein, TIMP-2 proteins are often obtained as misfolded, biologically inactive molecules with three-dimensional structures different from native protein, which may be due to the presence of incorrect 6-disulfide bridges [14], [15]. Modified TIMP-2 proteins such as Ala+TIMP-2 [16] and TIMP-2 chimera protein with mosaic serine protease [17] have also been developed and revealed a profound multi-functional mechanism of TIMP-2. However, a lack of information persists concerning the mechanism of its anticancer effect *in vivo*. Recently, a TIMP-2 fusion protein with human serum albumin (HSA/TIMP-2) was produced with high yield from the yeast *Saccharomyces cerevisiae* and demonstrated an inhibitory effect against tumor growth [18]. Furthermore, we have demonstrated that HSA/TIMP-2 exhibits favorable pharmacokinetic properties in prostate tumor xenograft [19].

In this study, our goals were to investigate the precise role of HSA/TIMP-2 in anti-tumor activity associated with angiogenesis and further define *in vivo* molecular links between anti-angiogenesis and the modulation of MMP-2. Our results show that HSA/TIMP-2, which does not inhibit MMP-2 activity or MT1-MMP expression *in vivo*, acts to promote anti-tumor and anti-angiogenesis activity through the downregulation of MMP-2 expression. This study is the first report of the *in vivo* mechanism underlying anti-tumor activity and anti-angiogenesis associated with MMP-2 modulation via TIMP-2 protein.

## Materials and Methods

### Cell culture

Rat prostate cancer MAT-LyLu (MLL) cells were kindly supplied by JungHan Yoon (Hallym University, Korea) [20] and were cultured in DMEM/F12 (Lonza, Walkersville, MD, USA), containing 10% (v/v) fetal bovine serum (Hyclone, Logan, UT, USA) with 100,000 U/L penicillin and 100 mg/L streptomycin (Gibco BRL, Grand Island, NY, USA). To generate a luciferase-expressing, stable MLL cell line, MLL cells were stably transfected with a CMV promoter-driven firefly luciferase expression construct (pcDNA3.1-Luc, a gift from Dr. Dongmin Kang, Ewha University, Seoul, Korea) using Lipofectamine™ (Invitrogen Co., Carlsbad, CA, USA). Cells were grown under hygromycin B (Sigma, St. Louis, MO, USA) selection, and positive clones were screened by bioluminescence, supplemented with 150 µg/mL d-luciferin (Biosynth International, Inc., Naperville, IL). Three clonal cell lines were initially selected, and the strongest bioluminescent derivative MLL cell line (MLL-Luc) was used in this study. The cell lines were monthly checked by PCR detection of mycoplasma contamination.

Human umbilical vein endothelial cells (HUVECs) were provided by Dr. Kwon Soo Ha (Kangwon University, Korea) [21] and cultured in growth medium 199 (Gibco-BRL), supplemented with 20% fetal bovine serum, 10 ng/mL human basic fibroblast growth factor (bFGF; R&D Systems Inc., Minneapolis, MN, USA), 100 U/ml penicillin, 100 µg/ml streptomycin and 5 U/ml heparin. Confluent HUVECs (passages 4–7) were used for the experiments. All cell lines were cultured at 37°C in an atmosphere of 5% CO_2_.

### In vitro and in vivo MMP-2 enzyme activity assay

MMP-2 activity was measured using a sensitive MMP-2 near-infrared fluorescent (NIRF) probe as a MMP-2 substrate [22]. For *in vitro* assay, inactive proMMP-2 (R&D Systems) was converted into the active form by incubation with 2.5 mM *p*-aminophenyl mercuric acid (Sigma) at 37°C for 30 min prior to use. HSA/TIMP-2, MMP inhibitor (MMP I, Calbiochem, San Diego, USA), or TIMP-2 were incubated in the reaction buffer (100 mM Tris, 5 mM CaCl_2_, 200 mM NaCl, and 0.1% Brij) containing 14 nM MMP-2 NIRF probe and 14 nM activated MMP-2 for 40 min. Changes in fluorescence intensity were measured using an IVIS-200 imaging system (Xenogen Corporation, Alameda, CA).

*In vivo* MMP-2 enzymatic activity was analyzed according to the method of Bremer *et al.* [23], with some modifications. Briefly, when MLL tumor had grown to about 500 mm^3^ in volume, MMP I (50 nM), HSA/TIMP-2 (80 mg/kg), or PBS control was intraperitoneally (ip) injected twice per day. Subsequently, MMP-2 NIRF probe (100 nM) was intravenously administered 30 min after the second injection of MMP I or HSA/TIMP-2. NIRF images were obtained 2 h after probe injection with Xenogen Living Image® software (Xenogen Corporation) as described in a previous study [19].

### Zymographic analysis

MMP-2 activity was analyzed by gelatin zymography according to the method of Uemura *et al.* [24], with some modifications. Briefly, HUVECs were grown to 70–80% confluence, washed once with PBS, and incubated for 72 h in serum-free medium in the presence of HSA/TIMP-2. The culture medium was centrifuged (1,000 rpm, 5 min), and the supernatants were subjected to gelatin zymography. Aliquots of the supernatants (10 µL) were mixed with sample buffer and applied directly to a 10% polyacrylamide gel containing 1 mg/mL gelatin and 0.1% (w/v) SDS. After electrophoresis, the gels were soaked in renaturing solution for 60 min and then incubated in developing solution overnight for maximum sensitivity. The gel was stained with 0.5% Coomassie blue G-250 (Thermo Scientific Co., Waltham, MA) for 30 min and destained until the bands could be seen clearly.

### Semi-quantitative analysis of mRNA levels

Total RNA and cDNA were prepared as described in our previous study [25]. PCR was performed with 100 ng cDNA per sample for MMP-2 and the housekeeping gene glyceraldehyde-3-phosphate dehydrogenase (GAPDH) to normalize the samples using Taq DNA polymerase (Invitrogen) in a PCR machine (PC-808, Astec, Japan). Primer sequences for human GAPDH were 5′-AGAAGGCTGGGGCTCATTTG-3′ (sense) and 5′-GGGGCCATCCACAGTCTTC-3′ (antisense), and the PCR for GAPDH was performed for 5 min at 95°C followed by 27 cycles of 30 s at 94°C, 30 s at 56°C, and 1 min at 72°C as well as a final elongation step for 10 min at 72°C. MMP-2 primers were 5′-GTGCTGAAGGACACACACTAAAGAAGA-3′ (sense) and 5′-TTGCCATCCTTCTCAAAGTTGTAGG-3′ (antisense) [26]. PCR conditions for MMP-2 were the same as described for GAPDH except for the primer annealing temperature (58°C) and the number of cycles (28 cycles). The amplification products of the PCR were electrophoresed on a 1% agarose gel and visualized by ethidium bromide staining. For quantification, the gels were scanned by an imaging system (TFX-20M, Vilber-Lourmat, France) and analyzed using the ImageJ program (open source ImageJ software available at http://rsb.info.nih.gov/ij/).

### Subcutaneous primary solid tumor model

Male Balb/c nude mice (Orient, Gyeonggi-do, Korea), 8–10 weeks old, were anesthetized by exposure to 1–3% isoflurane/O_2_ and administered 1×10^5^ MLL or MLL-Luc cells suspended in 50 µL sterile PBS by subcutaneous (sc) injection into the dorsal flank. Tumor growth was monitored for 5 days after injection by *in vivo* bioluminescence imaging (BLI) and external caliper measurements. Tumor volume was estimated by the formula, L×W^2^/2 (L = tumor length, W = tumor width). The use and care of animals was reviewed and approved by the Institutional Animal Care and Use Committee of the Korea Basic Science Institute (KBSI-AEC 1110).

### Bioluminescence imaging

*In vivo* BLI was performed with an IVIS-200 imaging system (Xenogen Corporation). Mice received the substrate D-luciferin by ip injection at a dose of 150 mg/kg dissolved in Dulbecco's PBS. At 10 min after injection, mice were anesthetized and then placed onto a warmed stage. Imaging was performed for 1–3 min, depending on the tumor size and time point. Generally, five mice were imaged at one time. Regions of interest in the tumor were quantified as photon flux (p/s) using Xenogen Living Image® software.

### Chick chorioallantoic membrane (CAM) assay

Anti-angiogenic activity was determined using a CAM assay as described previously [27]. Fertilized chicken eggs were incubated at 38°C in an 80% humidified atmosphere. On day 8, filter disks (0.5 cm in diameter) were loaded on the CAM. HSA/TIMP-2 (1 mg/dose) or the equivalent volume of PBS was deposited in the center of the filter. Treatment was repeated daily for 3 days, and CAM results were analyzed on the fourth day. Photos of each CAM were taken under a stereomicroscope (Stemi 2000-C, Carl Zeiss, Oberkochen, Germany) using a digital camera (G5 PC1049, Canon, Japan). Angiogenesis was quantified by counting the number of blood vessel branch points surrounding the filter disk; at least 10 viable embryos were tested for each treatment.

### Dynamic in vivo imaging of blood vessels

Images of peritumoral blood vessels in living prostate cancer xenografts were obtained using intravital fiberoptic confocal fluorescence microscopy (FCFM; Cellvizio® system; Mauna Kea Technologies, Paris, France) as described previously [28] and analyzed with ImageCell software version 3.6.1 (Mauna Kea Technologies). The maximum signal observed within each frame was averaged across a large set of representative frames (minimum 10) to determine the fluorescence reading for each mouse peritumoral blood vessel. For quantification, randomly selected frames were inspected blindly under an LSM 5 Pascal confocal fluorescent microscope (Carl Zeiss) using a fluorescein isothiocyanate (FITC) filter at 488 nm. The image was then analyzed using Pascal imaging software (version 2.8 SP1) to measure the mean fluorescent vascular density.

### Immunohistochemical analyses

After euthanizing the mice 15 days after 80 mg/kg HSA/TIMP-2 administration to prostate cancer xenografts, tumor tissues were collected and fixed overnight in freshly prepared 4% paraformaldehyde in PBS. Immunohistochemical analyses were performed on 8-µm sections using monoclonal antibodies specific for Ki-67 (1∶500; Abcam, Cambridge, MA), active caspase-3 (1∶500; Abcam), or MMP-2 (1∶500; Santa Cruz Biotechnology, Santa Cruz, CA) as described previously [29]. Slides were developed in 3,3′-diaminobenzidine tetrachloride (Sigma) as a chromogenic substrate and then counterstained with Mayer's hematoxylin. Images were taken with a light microscope (Carl Zeiss) equipped with a digital camera (DP71, Olympus, Japan) connected to a PC monitor.

For immunofluorescence staining, the sections were stained with monoclonal antibodies specific for PECAM-1 (1∶50; BD Biosciences, Transduction Laboratories, San Diego, CA, USA), MMP-2 (1∶200, Santa Cruz Biotechnology), or MT1-MMP (1∶200; Millipore, Billerica, MA) and subsequently exposed to Alexa 488-labeled goat anti-rat antibody or 546-labeled goat anti-rabbit antibody, or 633-labeled goat anti-mouse antibody (1∶500; Molecular Probes, Eugene, OR, USA). Sections were counterstained with Hoechst 33342 (1∶3,000; Invitrogen). Fluorescence signals were analyzed using a confocal laser scanning microscope (LSM-5 and LSM System, ver. 3.98; Carl Zeiss).

### Western blot analysis

Western blot analysis was carried out as described previously [30], and protein concentrations were determined by a standard protein assay (Bio-Rad, Hercules, CA). The primary antibodies were specific for PECAM-1 (1∶200; Santa Cruz Biotechnology), MMP-2 (1∶1,000; Santa Cruz Biotechnology), MT1-MMP (1∶200; Millipore), or actin (1∶10,000; Sigma). Detection was carried out using horseradish peroxidase-conjugated IgG (1∶5,000; Santa Cruz Biotechnology) and visualized using the ECL assay kit (Amersham, Little Chalfont, Buckinghamshire, UK). The band intensities obtained by western blot analysis were determined using the ImageJ program.

### Statistical analysis

For all results, values are expressed as the mean ± standard error of the mean (SEM). Statistical analyses were performed using the Student's *t* test for comparisons of 2 groups and using one-way or two-way ANOVA (Graphpad Software Inc version 3.05, San Diego, CA, USA) for multigroup comparisons. Significance was set at *P*<0.05.

## Results

### Anti-tumor activity of HSA/TIMP-2 in prostate cancer xenografts

**Figure 1:**
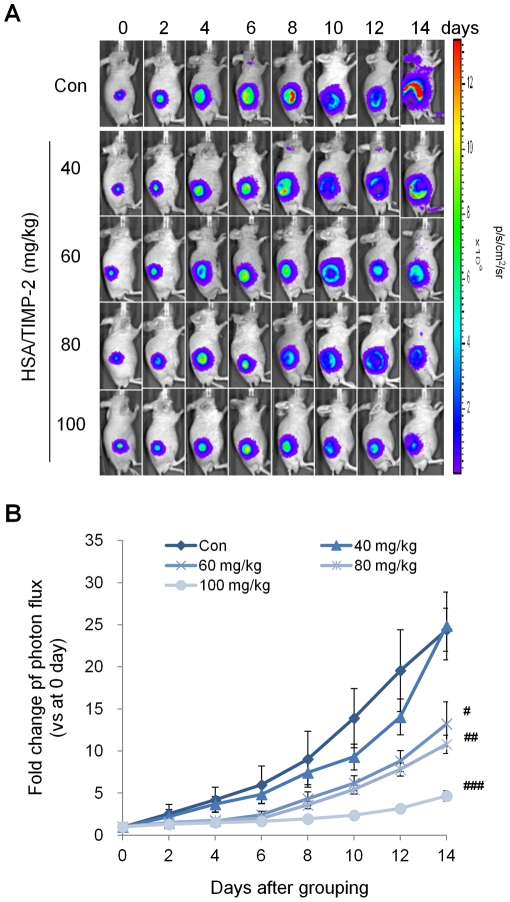
HSA/TIMP-2 inhibits tumor growth in MLL prostate cancer xenografts. (A) Representative optical images of prostate cancer xenografts. PBS as a control or HSA/TIMP-2 was ip injected every two days for 14 days, beginning 5 days after MLL-Luc cancer cell injection when the average tumor photon count had reached approximately 3.0×10^6^ p/s/cm^2^/sr. (B) Quantification of bioluminescence imaging. Data are expressed as the fold change of photon flux (p/s) compared with the control at day 0 and represent the mean ± SEM (*n* = 10 per group); ^#^ *P*<0.05 *vs.* control group on day 14 by one-way ANOVA followed by post-hoc Tukey's test. ^##^ *P*<0.01, ^###^ *P*<0.001.

To quantify the inhibitory effect of HSA/TIMP-2 on tumor growth with BLI, we used a luminescent cancer xenograft with a rat prostate cancer MLL cell line stably expressing firefly luciferase. HSA/TIMP-2 protein was ip injected into the MLL-Luc prostate cancer xenografts every other day for two weeks based on our previous pharmacokinetic results [19]. As shown in Fig. 1, bioluminescence signal reached 3.92±0.79×10^7^ p/s at day 14 after PBS injection in the control group, and significant inhibition of tumor growth was observed in the 60, 80, and 100 mg/kg HSA/TIMP-2 treatment groups: 45.9%, 55.8% and 80.8%, respectively, as compared to control (Fig. 1B; F_(4.45)_ = 13.69, *P*<0.0001, one-way ANOVA).

### Anti-proliferation, no apoptosis effect of HSA/TIMP-2

**Figure 2:**
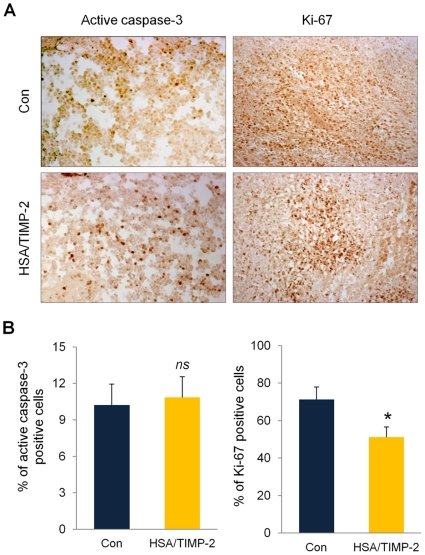
HSA/TIMP-2 inhibits tumor cell proliferation but not apoptosis in prostate tumors. (A) Representative immunohistochemical images of Ki-67 and active caspase-3 immunohistochemistry. Tumor sections were obtained at 15 days after initial injection of 80 mg/kg HSA/TIMP-2 (*n* = 4 per group). The representative images were taken at an original magnification of 200×. (B) Quantification of Ki67- and active caspase-3-positive cells as expressed by the percentage of brown pixels per field. The quantitative data represent the mean ± SEM (*n* = 4 per group). Significant difference compared to the control group: ^*^ *P*<0.05 by Student's *t* test. *ns* = non-significant.

Anti-tumor activity typically requires the coordinated action of tumor cell anti-proliferation and apoptosis [31]. Accordingly, to evaluate possible *in vivo* effects of HSA/TIMP-2 on apoptosis and proliferation, immunohistochemical staining data from treatment and control groups of tumor-bearing mice were compared. The percentages of active caspase-3-positive cells (apoptotic cells) in control and 80 mg/kg HSA/TIMP-2-treated groups were 10.2% and 10.8%, respectively, with no significant difference (Fig. 2B; *P* = 0.80 *vs.* control), suggesting no effect of HSA/TIMP-2 treatment on the apoptotic status of tumor cells. However, HSA/TIMP-2 treatment had a significant anti-proliferation effect in tumor cells *in vivo*. Strong immunostaining for Ki-67 was observed in tumors from the control group, whereas weak and moderate staining, with a 28.1% reduction in Ki-67 immunoreactivity, was found in the HSA/TIMP-2-treated group (Fig. 2B; *P*<0.05 *vs.* control). Similar to the anti-proliferation effect observed *in vivo*, decreased cell proliferation was also found in cultures of HUVECs (Fig. S1).

### In vivo anti-angiogenic activity of HSA/TIMP-2

**Figure 3:**
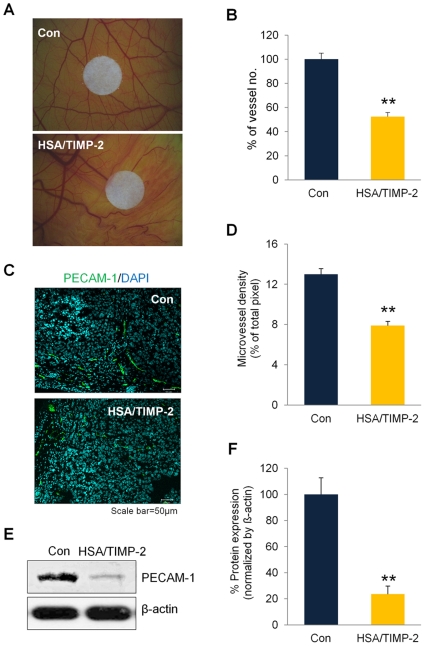
HSA/TIMP-2 inhibits angiogenesis in vivo. (A) Representative images of blood vessels in PBS (control, upper panel) or 1 mg/dose HSA/TIMP-2-treated CAM (lower panel). (B) Quantification of CAM angiogenesis. Blood vessel number was quantified in a circular perimeter surrounding the filter disk (*n* = 10 per group). (C) Representative image of PECAM-1 blood vessel visualized by immunofluorescence staining. The vascular density of control and HSA/TIMP-2-treated tumors was determined by staining frozen sections with anti-PECAM-1 antibody (green) followed by Hoechst nuclear counterstaining (blue). Scale bar = 50 µm. (D) Quantitative measurement of vascular density in the tissue sections determined by calculating the mean pixel intensity of PECAM-1 fluorescence staining per that of Hoechst nuclear staining. (E) *In vivo* PECAM-1 protein expression detected by western blot analysis of tumor lysates. (F) Quantification of band intensity normalized by ß-actin. All quantitative data except for CAM assay represents the mean ± SEM (*n* = 4 per group). Significant difference compared to the control group: ^**^ *P*<0.01 by Student's *t* test.

Although HSA/TIMP-2 has been shown to have an inhibitory effect on the formation of capillary-like phenotype in HUVEC cultures [18], [19], its direct contribution to *in vivo* anti-angiogenesis is not known. Thus, we performed CAM assays to investigate the anti-angiogenic activity of HSA/TIMP-2, which demonstrated that vascularization of the HSA/TIMP-2-treated group was significantly decreased by 47.7% compared with the control group (Fig. 3A, B; *P*<0.01). In analyzing tumor vascularization by immunofluorescence staining for endothelial cell marker PECAM-1, the microvessels of HSA/TIMP-2-treated tumor tissue demonstrated a significant 39.2% decrease in PECAM-1 level compared with the control group (Fig. 3C, D; *P*<0.01). Western blot analysis also revealed an even more severe 76.3% reduction in the expression of PECAM-1 in the HSA/TIMP-2-treated group (Fig. 3E, F; *P*<0.01 *vs.* control). Consistent with the observed decrease in PECAM-1 level, dynamic *in vivo* imaging of the vasculature using FCFM revealed that the relative FITC-stained vessel density significantly decreased by 50.8% in HSA/TIMP-2-treated group (Fig. S2; *P*<0.01 *vs.* control, Video S1 and S2).

### Regulation of MMP-2 and MT1-MMP expression by HSA/TIMP-2

**Figure 4:**
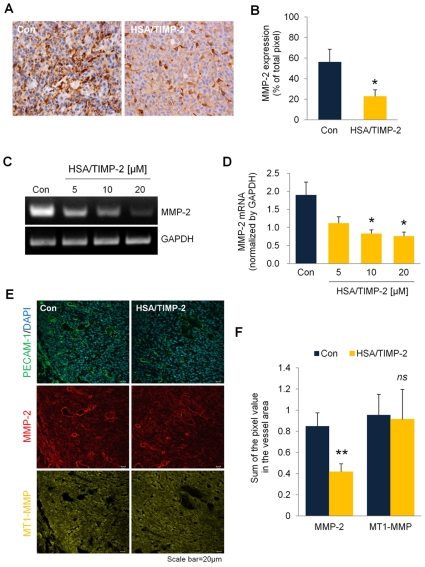
HSA/TIMP-2 inhibits MMP-2 expression independent of MT1-MMP expression. (A) Representative immunohistochemical images of MMP-2. The images were taken at an original magnification of 200×. (B) Quantification of MMP-2 expression determined by the percentage of brown pixels per field. (C) Representative RT-PCR images of MMP-2. HUVECs were treated with the indicated doses of HSA/TIMP-2 (lanes 2–4), and RNA was isolated 48 h after treatment. (D) Quantification of band intensity normalized by GAPDH. The quantitative data represent the mean ± SEM of duplicate samples from three independent experiments. (E) Representative images of PECAM-1, MMP-2 and MT1-MMP detected by immunofluorescence staining. Frozen sections of tumor tissues were triple-immunostained with anti-PECAM-1, anti-MMP-2 and anti-MT1-MMP followed by Hoechst nuclear (DAPI, blue) counterstaining. Scale bar = 20 µm. (F) Quantitative measurement determined by calculating the mean pixel intensity of MMP-2 (red) or MT1-MMP (green) fluorescence staining per that of Hoechst nuclear staining (blue). All quantitative data represents the mean ± SEM (*n* = 4 per group). Significant difference compared to the control group: ^**^ *P*<0.01, ^*^ *P*<0.05 by Student's *t* test. *ns* = non-significant.

Given the *in vivo* anti-angiogenic activity of HSA/TIMP-2, we explored the regulatory effects of HSA/TIMP-2 on MMP-2, a key regulator of tumor angiogenesis known to be modulated through an interaction with TIMP-2 [32], [33]. A similar decrease in MMP-2 was also revealed in the lysates of HSA/TIMP-2-treated tumors by western blot analysis (Fig. S3A, B). In addition, RT-PCR analysis showed that HSA/TIMP-2 treatment decreased MMP-2 mRNA in a dose-dependent manner in HUVECs (Fig. 4C, D).

To further identify whether the downregulation of MMP-2 in endothelial cells is associated with MT1-MMP, which facilitates peri/extracellular pro-MMP-2 activation, we performed triple-fluorescence immunostaining for PECAM-1, MMP-2 and MT1-MMP on tumor tissue. Quantitative analysis indicated that the level of MT1-MMP in PECAM-1-positive blood vessels did not significantly differ between control and HSA/TIMP-2-treated tumors (*P* = 0.918), which was confirmed by western blotting of tumor lysates from the HSA/TIMP-2-treated group (Fig. S3C, D; *P* = 0.096). In contrast, consistent with previous results, MMP-2 level in PECAM-1-positive blood vessels was significantly decreased by 50.9% in the HSA/TIMP-2-treated group (Fig. 4E, F; *P*<0.01 *vs.* control), indicating that inhibition of MMP-2 by HSA/TIMP-2 is independent of MT1-MMP expression.

### Effect of HSA/TIMP-2 on MMP-2 proteolytic activity in HUVECs

**Figure 5:**
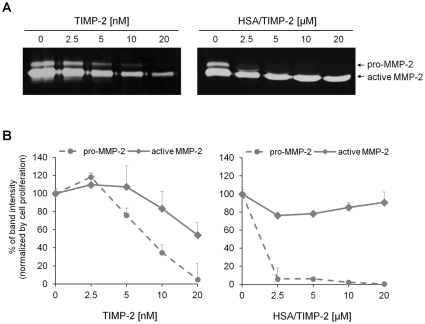
HSA/TIMP-2 inhibits extracellular proteolytic activity of MMP-2. (A) Representative images of the gelatin zymographic analysis. Culture media from HUVECs treated with TIMP-2 or HSA/TIMP-2 for 72 h were analyzed on a gelatin gel. Two forms of MMP-2 (latent form, 72 kDa; activated form, 62 kDa) are presented. (B) Quantification of band intensity. Data are expressed as the percentage of band intensity normalized with respect to the proliferation level (Fig. S4). The quantitative data represent the mean ± SEM of duplicate samples from three independent experiments.

The unexpected results on MT1-MMP expression led us to investigate the modulation of MMP-2 activity. We compared the effects of TIMP-2 and HSA/TIMP-2 on MMP-2 activity using zymographic analysis of HUVEC-conditioned media. As shown in Fig. 5A, TIMP-2 dose-dependently inhibited both pro-MMP-2 (72 kDa) and active MMP-2 (68 kDa) activity. However, the effect of HSA/TIMP-2 on MMP-2 activity was represented by distinct patterns in pro-MMP-2 activation. Pro-MMP-2 showed an outright loss of activity even at the low dose of HSA/TIMP-2 (2.5 µM), showing a dramatic decrease of 93.9% compared to that of control. Active MMP-2 activity was suppressed by HSA/TIMP-2 treatment, which had a maximal effect of 23.8% inhibition at 2.5 µM and did not decrease further at higher doses of HSA/TIMP-2 (Fig. 5B). This finding could be the result of unleashing endogenous MT1-MMP to promote pro-MMP-2 activation. This data support the *in vivo* evidence that HSA/TIMP-2 inhibited tumoral angiogenesis through downregulated expression of MMP-2, but not the modulation of MT1-MMP activity.

### Lack of the effect of HSA/TIMP-2 on MMP-2 activity

**Figure 6:**
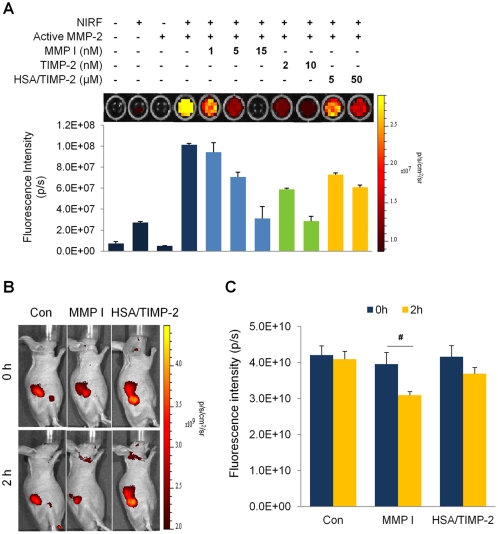
HSA/TIMP-2 does not inhibit MMP-2 activity. (A) *In vitro* MMP-2 inhibitory activity was measured by co-incubating with a MMP-2 NIRF probe and activated MMP-2 in the presence of TIMP-2, MMP-2 inhibitors, or HSA/TIMP-2, followed by fluorescence imaging with an IVIS-200 imaging system (*top*). The corresponding fluorescence intensity (p/s) was quantified in each sample from 3 independent experiments (*bottom*). A reaction containing NIRF and MMP-2 was used as a positive control in this experiment (lane 4). (B) Representative whole-body NIRF images. NIRF imaging of MLL tumor-bearing mice was performed at 2 h after injection of the MMP-2 NIRF probe in the presence of MMP I (*n* = 4), HSA/TIMP-2 (*n* = 5), or control PBS (*n* = 6). (C) The quantitation of fluorescent signal in the tumor. The fluorescence intensity (p/s) was quantified based on the total photon count determined from regions of interest. Each data point represents the mean ± SEM; ^#^ *P*<0.05 *vs.* control group by two-way ANOVA followed by Bonferroni post-hoc test.

The MMP-2 inhibitory activity of TIMP-2 is known to be lost by modification of the N-terminal of TIMP-2 such as carbamylation [34] or addition of an extra Ala [16], [35]. To determine whether the substitution of the TIMP-2 N-terminal with HSA alters TIMP-2 inhibitory activity, we compared the activity of HSA/TIMP-2 with TIMP-2 and an MMP inhibitor (MMP I) using a near-infrared fluorescent (NIRF) probe [22]. As shown in Fig. 6A, the NIRF probe demonstrated a strong fluorescent intensity (1.01±0.01×10^8^ p/s) after proteolytic activation by MMP-2, and this intensity was reduced by 75.9% with 10 nM TIMP-2 and by 69.4% with 15 nM MMP I pre-treatment as compared to non-treatment. However, 30 nM HSA/TIMP-2 treatment did not decrease NIRF signal (data not shown), and high doses of HSA/TIMP-2 barely inhibited NIRF signals by 18.2% at 5 µM and 39.9% at 50 µM compared to non-treatment. Thus, the inhibitory activity of HSA/TIMP-2 on MMP-2 was 4 orders of magnitude less than that of native TIMP-2 and MMP I. Furthermore, consistent with *in vitro* results, 2 h after injection of NIRF probe, HSA/TIMP-2-treated MLL-tumor xenografted mice demonstrated no NIRF intensity change, whereas the MMP I-treated group demonstrated NIRF intensity significantly decreased by 21.7% compared with the control group (Fig. 6C; F_(1,24)_ = 5.43, *P*<0.05, two-way ANOVA), providing further support for the loss-of-function effect of HSA/TIMP-2 on MMP-2 proteolytic activity.

## Discussion

In this study, we provide *in vivo* evidence for anti-tumor activity linked to anti-angiogenesis by HSA/TIMP-2 and further clarify that the anti-angiogenesis is associated with attenuated MMP-2 expression independent of MMP-2 activity as demonstrated by the absence of effects on MT1-MMP expression and pro-MMP-2 activation.

Our data concerning the effects of HSA/TIMP-2 on *in vivo* angiogenesis are in agreement with the earlier findings of a null-MMP inhibitor form of TIMP-2 (Ala+TIMP-2) [16]. HSA/TIMP-2 has no inhibitory effect on MMP-2 activity *in vitro* and *in vivo*; nevertheless HSA/TIMP-2 demonstrated a prominent anti-proliferation effect on tumor cells in cancer xenografts. HSA/TIMP-2 also interferes with processes critical to angiogenesis such as endothelial cell proliferation and capillary-like tube formation [19], and thus shows *in vivo* anti-angiogenesis effects. Additionally, we have verified that the anti-angiogenic activity is differentially modulated by HSA/TIMP-2 treatment leading to downregulation of MMP-2 transcript in HUVEC cells. This finding is consistent with previous reports that MMP-2 deficiency by genetic ablation or siRNA-based approaches results in reduced angiogenesis [36], [37], [38]. Interestingly, however, HSA/TIMP-2 did not induce downregulation of MT1-MMP expression *in vivo*. In the zymographic analysis, we found no inhibitory action of HSA/TIMP-2 on pro-MMP-2 activation, resulting in the sustained activity of active MMP-2. These results suggest a disconnection between anti-angiogenic activity and MMP-2 activity. This concept is supported by our results showing no effect of HSA/TIMP-2 on MT1-MMP expression and other findings concerning the MMP-2-independent anti-angiogenic activity of TIMP-2 [16], [39]. Moreover, recent studies have demonstrated that the C-terminal domain of TIMP-2, in particular loop 6, inhibits endothelial cell proliferation and angiogenesis [40], suggesting a potential role of the C-terminal domain of TIMP-2 in the anti-angiogenic activity of HSA/TIMP-2.

Cancer development involves complex multistep processes, but all cancers share the ability to proliferate beyond the constraints limiting growth in normal tissue and to resist the intrinsic property of self-destruction called apoptosis [41], [42]. Because deregulated cell proliferation together with suppressed cell death provide a common platform for tumor progression, they present targets for cancer therapy [43]. Numerous studies have considered to these two defects as cancer targets, whereas the effects of TIMPs on cell proliferation or apoptosis have been restricted to cell-based experiments and elicited divergent effects even in the same cell types [44], [45], [46], [47]. Consistent with previous observations in melanoma cells and human microvascular endothelial cells [47], [48], [49], we demonstrated that cell proliferation was potently downregulated in HSA/TIMP-2-treated tumor tissue as well as HUVECs. In contrast, the effect of HSA/TIMP-2 on apoptosis did not differ from control, which is contrary to the MMP-independent proapoptotic effect of TIMP-2 described previously [39], [50], [51]. This difference may be due to the differing ability of HSA/TIMP-2 and TIMP-2 to regulate the expression of MMP-2. With respect to the interaction of death pathways with MMP-2, Shapiro *et al.* [52] demonstrated that active MMP-2 induces apoptosis of endothelial cells via the p38 MAPK apoptotic pathway, which enhances MMP-2 synthesis and its activation probably through MT1-MMP. In contrast, Preaux *et al.* [53] showed that apoptosis promotes pro-MMP-2 activation through increased MT1-MMP expression. The relationship between apoptosis and MMP-2 is controversial; however, our data support the hypothesis that downregulation of MMP-2 expression by HSA/TIMP-2 is associated with an attenuation of apoptosis.

Our finding that high doses of HSA/TIMP-2 protein inhibit angiogenesis and subsequently inhibit tumor growth is consistent with the findings of previous TIMP-2 studies [10], [11], [16], [54]. However, at lower doses (less than 40 mg/kg), HSA/TIMP-2 demonstrated a stimulative effect on prostate cancer xenografts (data not shown). This difference may be explained by the bi-functional activity of TIMP-2 in angiogenesis, as proposed by Seo *et al.* [16]: low levels of TIMP-2 enhance angiogenesis by the activation of MMPs through an MT1-MMP-dependent mechanism, whereas high levels of TIMP-2 downregulate angiogenic signaling through binding to α_3_β_1_ integrin. Thus, our determination of the effective dose for anti-tumor activity provides important information for cancer therapies aimed at modulating angiogenesis.

In agreement with a previous study of B16BL6 melanoma xenografts [18], systemic administration of HSA/TIMP-2 protein also demonstrated a potent anticancer effect in MLL prostate cancer xenografts. However, the minimum effective doses of the two studies differ slightly. It was determined as 60 mg/kg in this study, whereas tumor growth was suppressed at 40 mg/kg in the B16BL6 melanoma xenograft model. This difference could be explained by different experimental methods such as the number of drug injections, the cancer cell line used for tumor xenografts, and the detection method of tumor growth. In this study, we used BLI analysis with a MLL bioluminescent xenograft model [55], which has several advantages over traditional caliper measurements including accurate monitoring of tumor growth as well as earlier tumor detection as the tumor progresses. Traditional volumetric analysis by caliper measurements inevitably counts necrotic areas and edema as well as cancer cells in a tumor, but the bioluminescence signals of BLI analysis are derived from only viable cancer cells and thus exclude a systematic bias from the inclusion of necrotic areas and edema [56], [57]. This different sensitivity of detection methods may explain the different minimum effective doses of the two studies, as a moderate correlation was observed between tumor volume and photon flux (R^2^ = 0.89, Fig. S5). In addition to the detection method of tumor growth, another explanation is the number of HSA/TIMP-2 administrations. HSA/TIMP-2 was injected every two days in this study, whereas 40 mg/kg HSA/TIMP-2 was injected daily in the B16BL6 melanoma xenograft study [18].

In conclusion, this study is the first to report that HSA/TIMP-2 has dose-dependent effects on tumor growth in male Balb/c nude mice, highlighting the importance of minimum effective dose determination on anti-tumor activity *in vivo*. We also demonstrate that the anti-tumor activity of HSA/TIMP-2 is strongly associated with anti-angiogenesis attributed to a reduction of MMP-2 production but without inhibition of MT1-MMP, indicating that HSA/TIMP-2 plays a more complex role in anti-angiogenesis. Taken together, these findings provide a useful basis for the *in vivo* mechanistic study of TIMP-2 anti-tumor activity and suggest that HSA/TIMP-2 holds promise as an anticancer drug.

## Supporting Information

Figure S1 **Effect of HSA/TIMP-2 on HUVEC proliferation.** HUVECs were stimulated with 50 ng/mL bFGF followed by treatment with 10 µM HSA/TIMP-2 for 48 h. Proliferation of viable cells was assessed by WST-1 assay (Takara, Kyoto, Japan). Results show the percentage of maximum proliferation obtained by stimulation of cells with bFGF alone, after correction for the basal rate of proliferation under serum-free conditions (100%). The quantitative data represent the mean ± SEM of triplicate samples from three independent experiments. Significant difference compared to the control group: ^**^ *P*<0.01 by Student's *t* test. ^***^ *P*<0.001.
(TIF)

Figure S2 **Inhibitory effect of HSA/TIMP-2 on angiogenesis in vivo.** (A) *In vivo* images of peritumoral blood vessels taken by FCFM imaging following intra-arterial injection of 75 mg/kg FITC-dextran. Representative images were captured at an original magnification of 20×. (B) The quantitative data from the mean area of vessels represents the mean ± SEM. Vessel area was determined as the area stained with FITC-dextran. The quantitative data represents the mean ± SEM (*n* = 4 per group). Significant difference compared to the control group: ^**^ *P*<0.01 by Student's *t* test.
(TIF)

Figure S3 **HSA/TIMP-2 does not inhibit MT1-MMP expression.** (A) Detection of MMP-2 (72 kDa) protein expression by western blot analysis of tumor lysates. (B) Quantification of band intensity normalized by ß-actin. (C) Detection of MT1-MP (54 kDa) protein expression by western blot analysis of tumor lysates. (D) Quantification of band intensity normalized by ß-actin. All quantitative data represent the mean ± SEM (*n* = 4 per group). Significant difference compared to the control group: ^*^ *P*<0.05 by Student's *t* test. *ns* = non-significant.
(TIF)

Figure S4 **Effect of TIMP-2 or HSA/TIMP-2 on HUVECs proliferation.** HUVECs (5×10^3^/well) were plated in 96-well plates and treated with TIMP-2 or HSA/TIMP-2 at the indicated concentration for 48 h. Proliferation of viable cells was assessed by WST-1 assay (Takara). Data represent the mean ± SEM of triplicate samples from three independent experiments.
(TIF)

Figure S5 **Correlation between BLI and tumor volume in vivo.** (A) Image of MLL-Luc prostate cancer xenografts. MLL-Luc cells (1×10^5^) were implanted into the dorsal flank by sc injection. (B) Quantification of bioluminescence intensity and tumor volume. BLI and caliper measurements (L×W^2^/2) were acquired at the indicated time point 5 days after implantation (*n* = 6). A moderate correlation between BLI (p/s, *y*-axis) and tumor volume (mm^3^, *x*-axis) was observed (R^2^ = 0.89).
(TIF)

Video S1 **Angiography of tumoral vessels.** Intravital fiberoptic confocal fluorescence microscopy of peritumoral vessels in a MLL prostate cancer xenograft. Tumoral vasculature is imaged after 75 mg/kg FITC-dextran injection (shown in green).
(MPEG)

Video S2 **Angiography in HSA/TIMP-2-treated tumoral vessels.** Multiphoton intravital fiberoptic confocal fluorescence microscopy of peritumoral vessels in a MLL cancer xenograft. Vasculature is imaged after 75 mg/kg FITC-dextran injection (shown in green).
(MPEG)
