# Supplementary figures and images for: TIMP-2 Fusion Protein with Human Serum Albumin Potentiates Anti-Angiogenesis-Mediated Inhibition of Tumor Growth by Suppressing MMP-2 Expression
Source: PLoS One. 2012 Apr 24;7(4):e35710. doi: 10.1371/journal.pone.0035710 (PMC3335789; doi:10.1371/journal.pone.0035710)

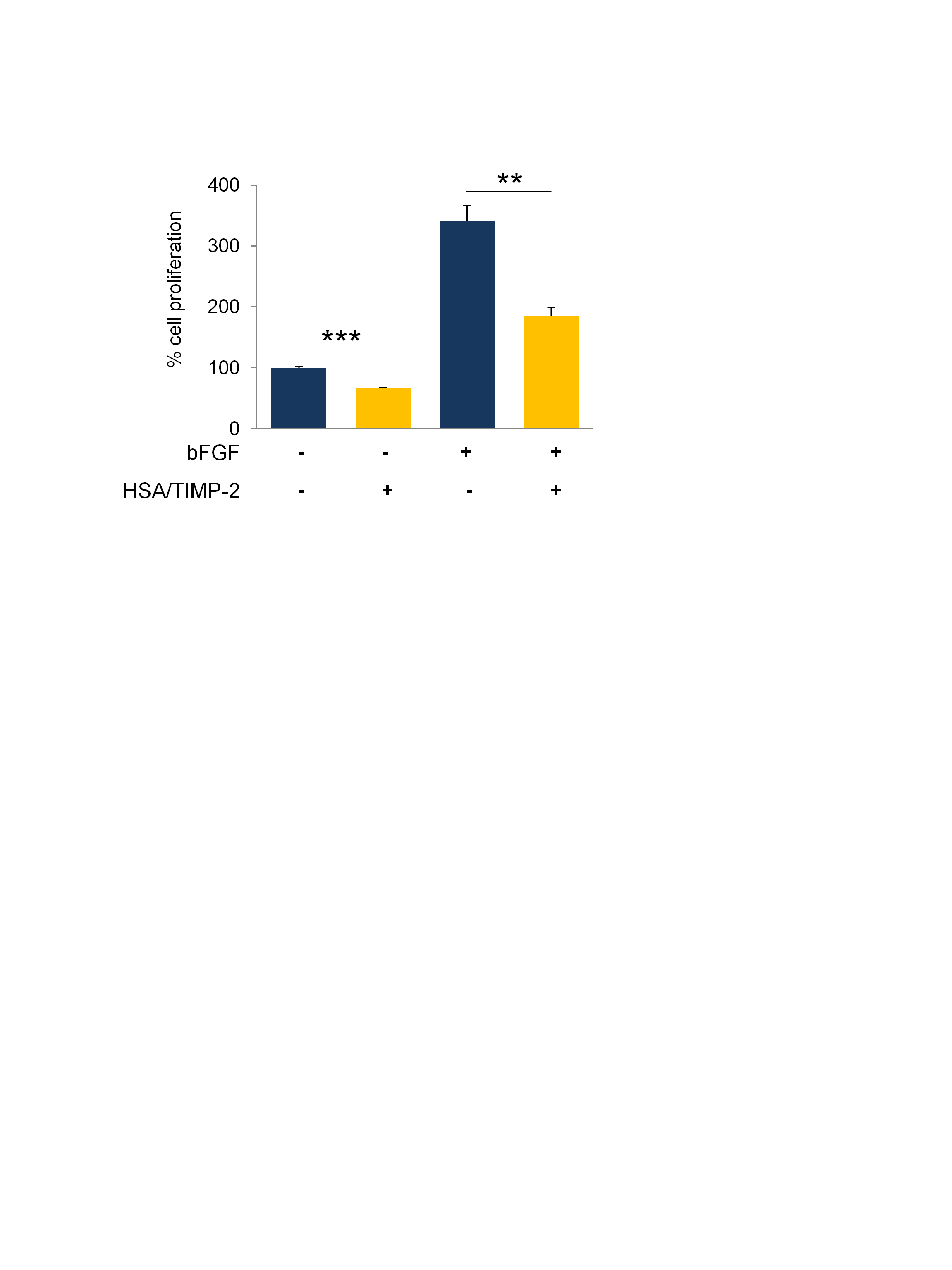

Supplement: Figure S1 — Effect of HSA/TIMP-2 on HUVEC proliferation. HUVECs were stimulated with 50 ng/mL bFGF followed by treatment with 10 µM HSA/TIMP-2 for 48 h. Proliferation of viable cells was assessed by WST-1 assay (Takara, Kyoto, Japan). Results show the percentage of maximum proliferation obtained by stimulation of cells with bFGF alone, after correction for the basal rate of proliferation under serum-free conditions (100%). The quantitative data represent the mean ± SEM of triplicate samples from three independent experiments. Significant difference compared to the control group: ** P<0.01 by Student's t test. *** P<0.001. (TIF) [file pone.0035710.s001.tif]

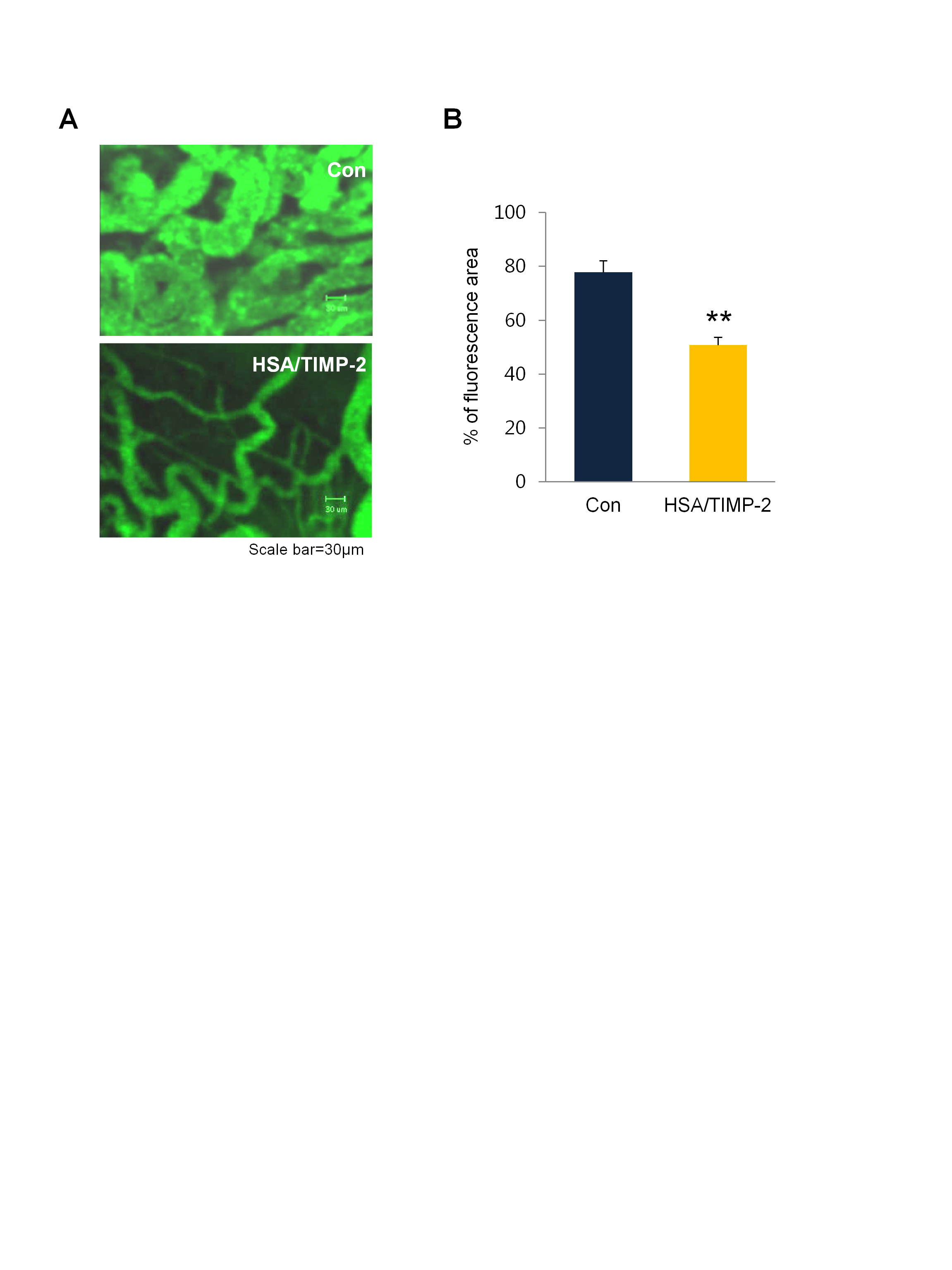

Supplement: Figure S2 — Inhibitory effect of HSA/TIMP-2 on angiogenesis in vivo. (A) In vivo images of peritumoral blood vessels taken by FCFM imaging following intra-arterial injection of 75 mg/kg FITC-dextran. Representative images were captured at an original magnification of 20×. (B) The quantitative data from the mean area of vessels represents the mean ± SEM. Vessel area was determined as the area stained with FITC-dextran. The quantitative data represents the mean ± SEM (n = 4 per group). Significant difference compared to the control group: ** P<0.01 by Student's t test. (TIF) [file pone.0035710.s002.tif]

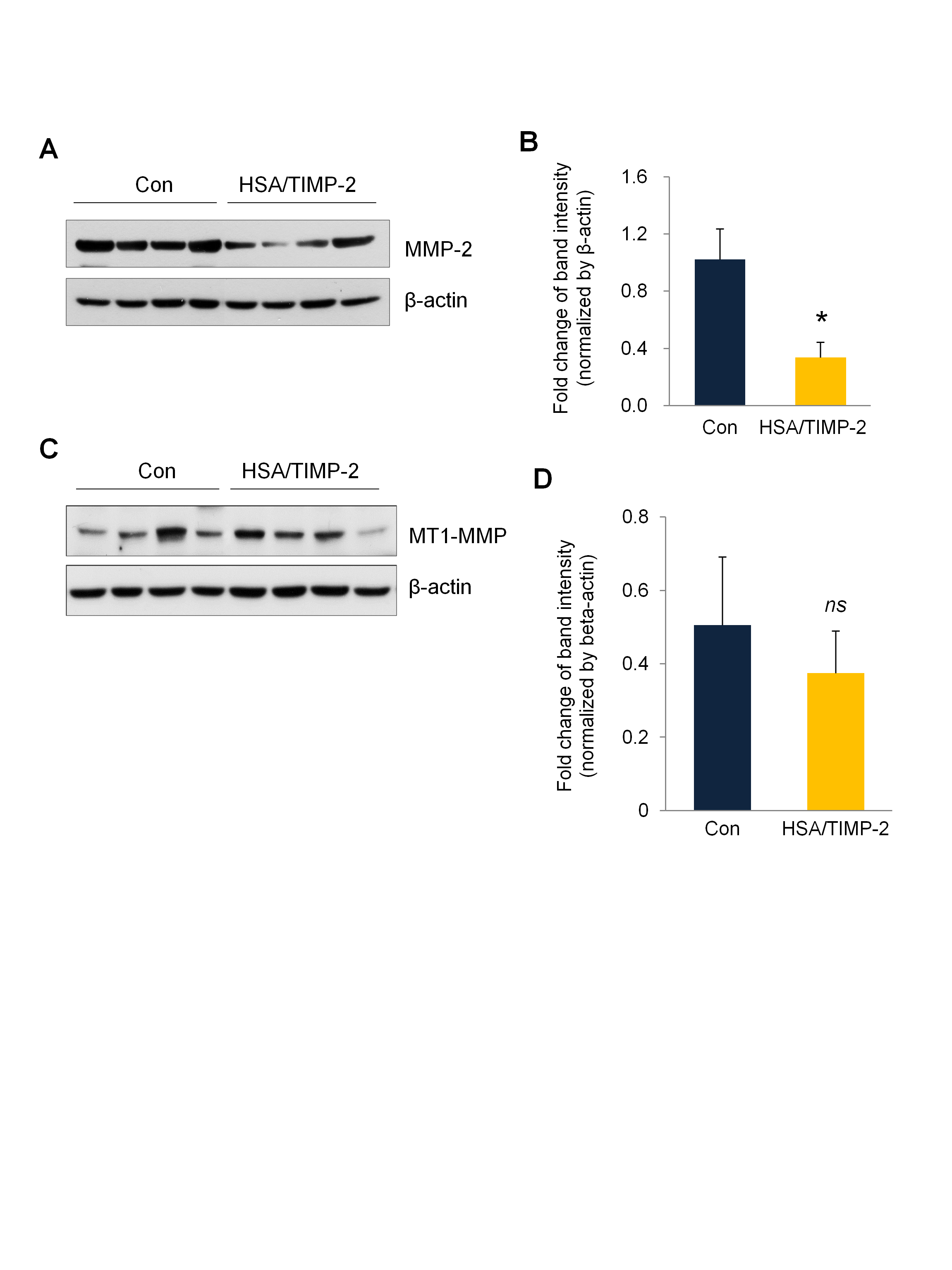

Supplement: Figure S3 — HSA/TIMP-2 does not inhibit MT1-MMP expression. (A) Detection of MMP-2 (72 kDa) protein expression by western blot analysis of tumor lysates. (B) Quantification of band intensity normalized by ß-actin. (C) Detection of MT1-MP (54 kDa) protein expression by western blot analysis of tumor lysates. (D) Quantification of band intensity normalized by ß-actin. All quantitative data represent the mean ± SEM (n = 4 per group). Significant difference compared to the control group: * P<0.05 by Student's t test. ns = non-significant. (TIF) [file pone.0035710.s003.tif]

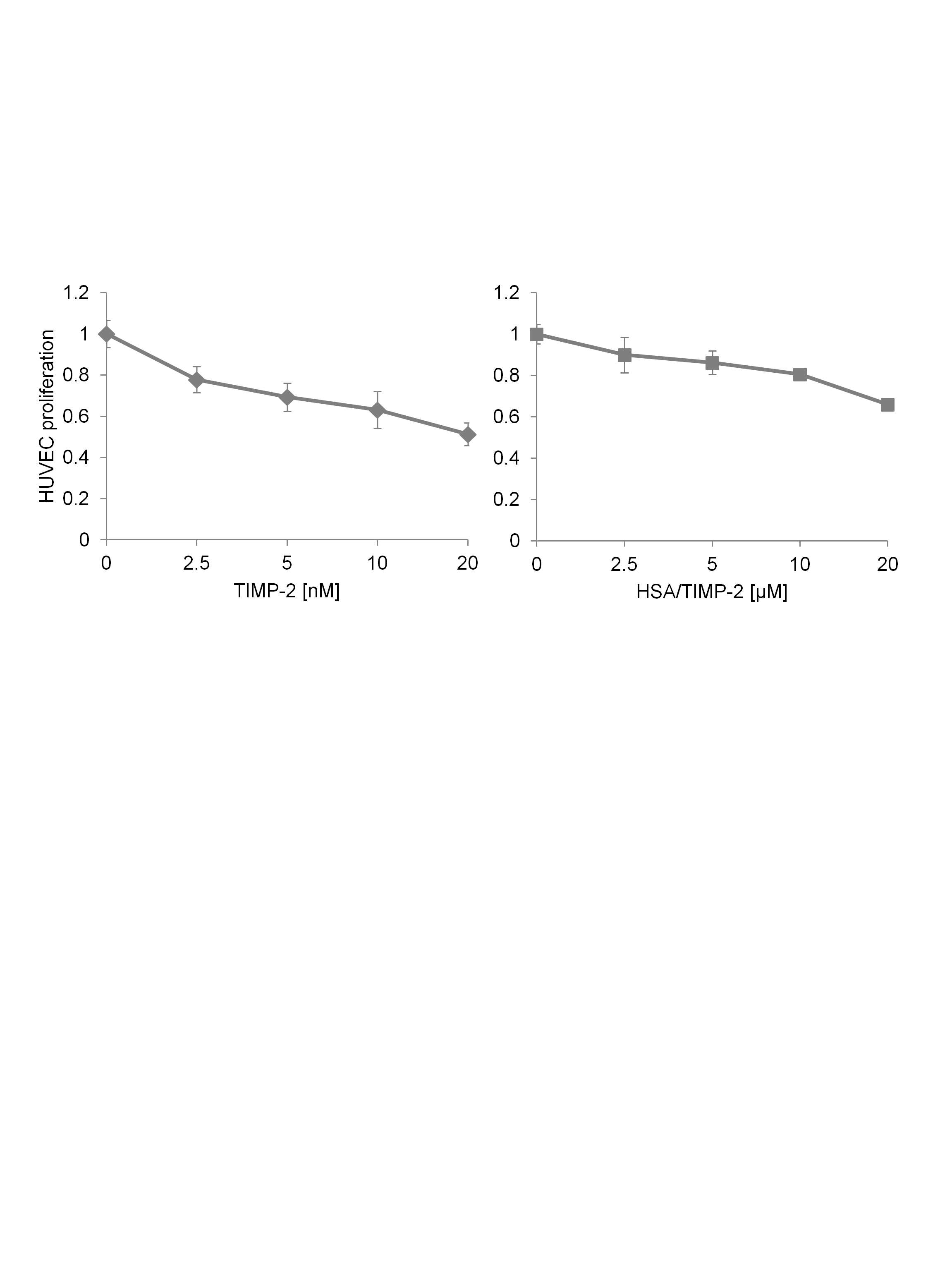

Supplement: Figure S4 — Effect of TIMP-2 or HSA/TIMP-2 on HUVECs proliferation. HUVECs (5×10^3^/well) were plated in 96-well plates and treated with TIMP-2 or HSA/TIMP-2 at the indicated concentration for 48 h. Proliferation of viable cells was assessed by WST-1 assay (Takara). Data represent the mean ± SEM of triplicate samples from three independent experiments. (TIF) [file pone.0035710.s004.tif]

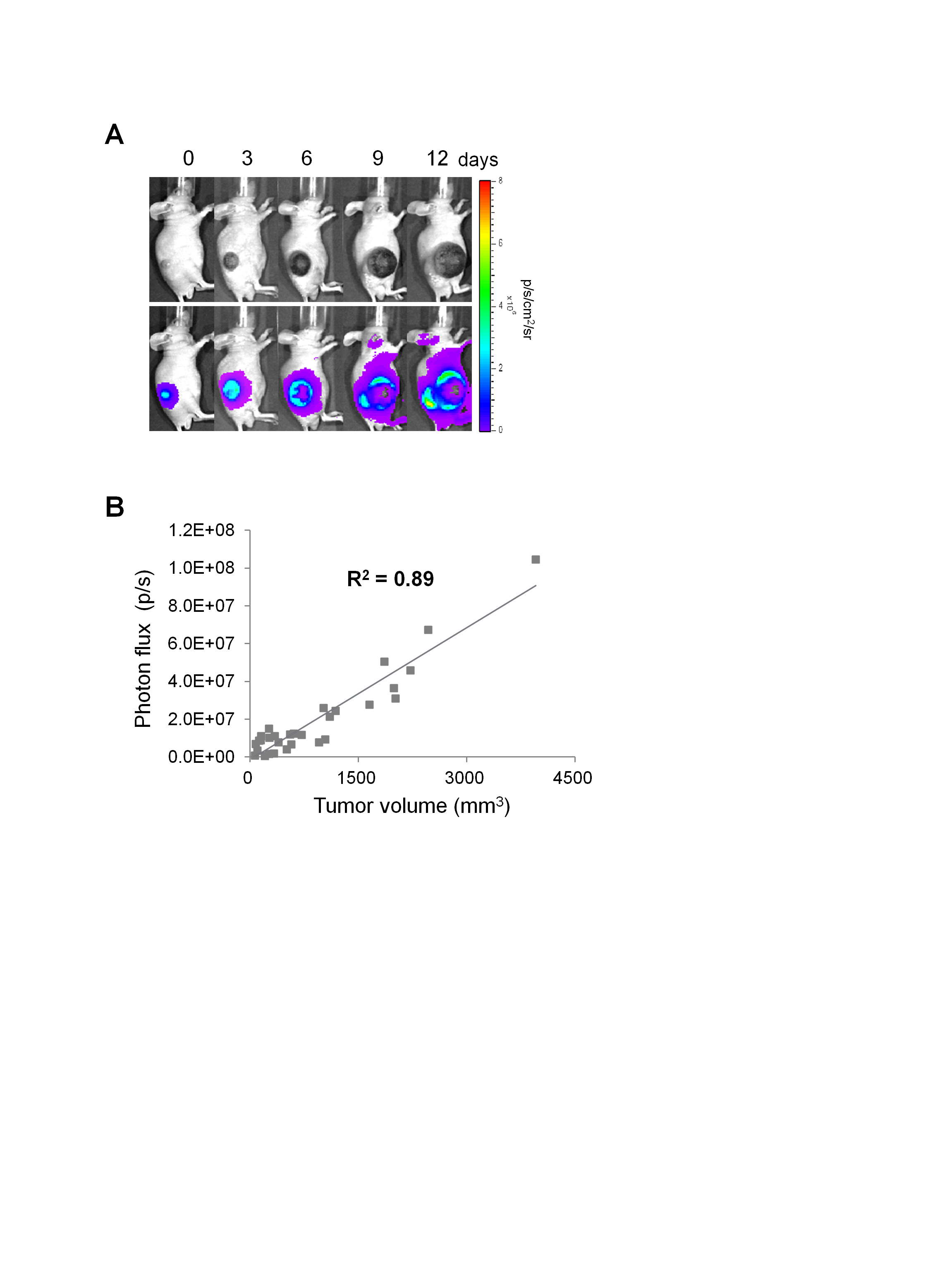

Supplement: Figure S5 — Correlation between BLI and tumor volume in vivo. (A) Image of MLL-Luc prostate cancer xenografts. MLL-Luc cells (1×10^5^) were implanted into the dorsal flank by sc injection. (B) Quantification of bioluminescence intensity and tumor volume. BLI and caliper measurements (L×W2/2) were acquired at the indicated time point 5 days after implantation (n = 6). A moderate correlation between BLI (p/s, y-axis) and tumor volume (mm3, x-axis) was observed (R2 = 0.89). (TIF) [file pone.0035710.s005.tif]
